# Inflammation, Senescence and MicroRNAs in Chronic Kidney Disease

**DOI:** 10.3389/fcell.2020.00739

**Published:** 2020-08-06

**Authors:** Andres Carmona, Fatima Guerrero, Maria Jose Jimenez, Francisco Ariza, Marisa L. Agüera, Teresa Obrero, Victoria Noci, Juan Rafael Muñoz-Castañeda, Mariano Rodríguez, Sagrario Soriano, Juan Antonio Moreno, Alejandro Martin-Malo, Pedro Aljama

**Affiliations:** ^1^Maimonides Biomedical Research Institute of Cordoba (IMIBIC), Reina Sofia University Hospital, Córdoba, Spain; ^2^Department of Medicine, University of Córdoba, Córdoba, Spain; ^3^Nephrology Unit, Reina Sofia University Hospital, University of Córdoba, Córdoba, Spain; ^4^Anesthesia Unit, Reina Sofía University Hospital, Córdoba, Spain; ^5^Spanish Renal Research Network (REDinREN), Institute of Health Carlos III, Madrid, Spain; ^6^Department of Cell Biology, Physiology and Immunology, University of Córdoba, Córdoba, Spain

**Keywords:** chronic kidney disease, microvesicles, monocytes CD14+CD16++, microRNAs, vascular smooth muscle cells

## Abstract

**Background:**

Patients with chronic kidney disease (CKD) show a chronic microinflammatory state that promotes premature aging of the vascular system. Currently, there is a growth interest in the search of novel biomarkers related to vascular aging to identify CKD patients at risk to develop cardiovascular complications.

**Methods:**

Forty-five CKD patients were divided into three groups according to CKD-stages [predialysis (CKD4-5), hemodialysis (HD) and kidney transplantation (KT)]. In all these patients, we evaluated the quantitative changes in microRNAs (miRNAs), CD14+C16++ monocytes number, and microvesicles (MV) concentration [both total MV, and monocytes derived MV (CD14+Annexin V+CD16+)]. To understand the molecular mechanism involved in senescence and osteogenic transdifferentation of vascular smooth muscle cells (VSMC), these cells were stimulated with MV isolated from THP-1 monocytes treated with uremic toxins (txMV).

**Results:**

A miRNA array was used to investigate serum miRNAs profile in CKD patients. Reduced expression levels of miRNAs-126-3p, -191-5p and -223-3p were observed in CKD4-5 and HD patients as compared to KT. This down-regulation disappeared after KT, even when lower glomerular filtration rates (eGFR) persisted. Moreover, HD patients had higher percentage of proinflammatory monocytes (CD14+CD16++) and MV derived of proinflammatory monocytes (CD14+Annexin V+CD16+) than the other groups. *In vitro* studies showed increased expression of osteogenic markers (BMP2 and miRNA-223-3p), expression of cyclin D1, β-galactosidase activity and VSMC size in those cells treated with txMV.

**Conclusion:**

CKD patients present a specific circulating miRNAs expression profile associated with the microinflammatory state. Furthermore, microvesicles generated by monocytes treated with uremic toxins induce early senescence and osteogenic markers (BMP2 and miRNA-223-3p) in VSMC.

## Introduction

Chronic inflammation is a common characteristic of patients with chronic kidney disease (CKD) that contributes to the development and progression of cardiovascular disease (CVD). In these patients, uremia-associated chronic inflammation plays a relevant role in endothelial cell damage (London, 2003; Cannata-Andía et al., 2006), coronary heart disease (Dregan et al., 2014) and premature aging of the vascular wall (Stenvinkel and Larsson, 2013; Carracedo et al., 2020).

microRNAs (miRNAs) are short, single-stranded and small no coding RNAs of approximately around 22 nucleotides in length that regulate gene expression (Leopold, 2014). Dysregulation of miRNAs has been linked to the pathophysiology of many inflammatory diseases, such as kidney and cardiovascular diseases (Metzinger-Le Meuth et al., 2019; Zhao et al., 2019). *In vivo* studies have reported that the expression of miR-126 is reduced in end stage renal disease and hemodialysis patients (Dziedzic et al., 2016; Brigant et al., 2017; Fourdinier et al., 2019). miR-126 is endothelial-specific and it is implicated in endothelial dysfunction promoting angiogenesis, endothelial proliferation and inhibiting vascular inflammation (Hu et al., 2015). Likewise, miR-223 may be involved in CKD development because of their role in inflammation and regulation of mineral metabolism (Ulbing et al., 2017). It has been reported that in the inflammatory context of CKD, miRNA-223 expression is increased in the aorta and conversely, the levels of this miRNA are decreased in serum (Taïbi et al., 2014). However, there are few works describing the circulating miRNAs in CKD and little is known about their possible biological and clinical impact in this process.

On the other hand, CD14+CD16++ monocytes are senescent immune cells (Ziegler-Heitbrock et al., 2010; Merino et al., 2011), that are involved in inflammatory response of CKD patients (Merino et al., 2008; Heine et al., 2012). These monocytes express high levels of integrins that allow the adherence of these cells to the endothelium and further infiltration to the vascular wall. Moreover, CD14+CD16++ cells have a high capacity to produce cytokines, angiogenic factors and microvesicles (MV) (Ramírez et al., 2011). MV are small vesicles (0.1–1 μm) released in response to activation of the cell, but also during stress and apoptosis (Yáñez-Mó et al., 2015). MV are commonly detected using flow cytometry to assess their antigenic characteristics and capacity to bind annexin V (Connor et al., 2010). MV are involved in promotion of vascular damage through different mechanisms, including increased coagulation (Diamant et al., 2004), inflammation (Carmona et al., 2017b; Guerrero et al., 2020) and cell-cell interactions (Ahn, 2005; Kanada et al., 2015). Plasma concentration of MV has been shown to be associated with cardiovascular risk (Baron et al., 2012), myocardial infarction (Morel et al., 2004) and CKD on hemodialysis (Carmona et al., 2017a). Altogether, CD14+CD16++ cells play a key role in upregulation of a chronic inflammatory response in elderly people and CKD patients, as well as a direct relationship with development of CVD.

In the present study, we determined quantitative changes of miRNAs, CD14+C16++ monocytes and MV in CKD patients at different disease stages. In a second step, we performed *in vitro* studies to analyze the role of monocytes-derived MV on VSMC senescence and osteogenic process.

## Materials and Methods

### Subjects

Forty-five CKD patients were selected from the Nephrology Unit, Reina Sofia University Hospital (Cordoba, Spain). Patients were divided into three groups according to stages of CKD: 17 patients in predialysis (CKD stages 4-5 without renal replacement therapy, CKD4-5), 11 patients on hemodialysis (stable for at least 12 months, HD) and 17 renal transplantation patients (<12-month kidney transplantation, mean time since transplantation 5 months, KT). Ten healthy elderly subjects (30% men) without CKD associated with aged (>85 years old) were included as controls. Elderly subjects have normal creatinine and blood urea nitrogen (BUN) levels (0.8 ± 0.1 mg/dl and 19.1 ± 2.9 mg/dl, respectively).

The study was approved by the Reina Sofia Hospital Ethics Committee and all subjects provided written informed consent. Authors state our adherence to the Declaration of Helsinki. Exclusion criteria were patients with active neoplasm, diabetes, active infection and positive viral markers as hepatitis B surface antigen (HBsAg) positive, anti HCV and HIV. Written informed consent was obtained from all patients included in this study.

### Blood Samples, Measurements and Assays

Blood samples were collected from a large antecubital vein using a 21-gauge needle directly into Vacutainer^®^ tubes (Becton Dickinson, United Kingdom). For each patient, 3 mL of blood was drawn from the venous line and collected in sodium citrate and serum vacutainer tubes. HD patient group the sample was collected just before the dialysis session. Dialysis system was free of bacteria (<100 colony-forming units per milliliter) and bacteriological contaminants (endotoxin levels < 0.025 endotoxin units) throughout the entire study.

The blood was centrifuged, within 30 min of collection, at 1500 × *g* for 10 min at room temperature (RT) to obtain platelet poor plasma (PPP) or serum. Aliquots for the measurement of microvesicles and miRNAs were stored at −80°C until to be use. Prior to analysis, samples were thawed on ice.

Haemoglobin levels were measured with an automatic analyzer (Abbott Cell-Dyn 4000; Abbott Laboratories, Abbott Park, IL, United States). The high-sensitivity C-reactive protein (CRP) levels were determined by immunoturbidimetry; the reagents were provided by Abbott Laboratories (Abbott Park, IL, United States). The levels of parathyroid hormone (PTH) were determined by the radioimmunoassay (RIA) method (Nichols Institute, The Netherlands). Glucose by RIA (Izotop Bioassays, Budapest, Hungary). Serum phosphate, creatinine and BUN were measured by spectrophotometry (Biosystems, Barcelona, Spain). Immunoturbidimetry (bromocresol purple) was used to measure serum albumin.

### RNA Isolation

Total RNA, including the miRNAs fraction, was extracted from serum using a comercial, column-based system, (miRCURY^TM^ RNA isolation kit – Biofluids, Exiqon, Vedbaek, Denmark) according to the manufacturer’s protocol with minor adjustments. Briefly, 200 μl serum was mixed with Lysis Solution BF containing 1 μg carrier-RNA per 60 μl Lysis Solution BF. Tube was vortexed and incubated for 3 min at RT. Spike-in solution containing non-mammalian synthetic miRNAs (ath-miR159a, cel-miR-248, cel-miR-254, osa-miR-414, osa-miR-442) was added to each sample followed by the addition of 20 μl Protein Precipitation Solution BF. Tube was vortexed, incubated for 1 min at RT and centrifuged to 11000 × *g* for 3 min. Clear supernatant was transferred into a new collection tube and 270 μl isopropanol was added. The solution was loaded onto binding column. Column was incubated for 2 min at RT and centrifuged for 30 s at 11000 × *g*. After washing, the dry column was transferred into a new collection tube and 20 μl RNase free water was added directly onto the membrane of the microRNA spin column. Column was incubated for 1 min at RT and centrifuged for 1 min at 11000 × *g*. Purified RNA quality was evaluated by spectrophotometer (Denovix DS-11) Isolated total RNA was stored at −80°C.

### RNA Expression Analysis

Analysis was performed on 12 samples, four random sampling of each group, using nCounter Analysis System (NanoString Technologies, Seattle, WA, United States) and the digital multiplexed nCounter^®^ human v3 miRNA Expression Assay which contains 827 unique miRNA barcodes. Probes for endogenous miRNAs for ath-miR159a, cel-miR-248, cel-miR-254, osa-miR-414 and osa-miR-442 are incorporated in NanoString codeSets and were used for analysis along with positive and negative controls. A total of 3 μl was used per sample and conditions were set according to the manufacturer’s recommended protocol. Subsequently, nCounter data files (RCC files) for each sample were imported into nSolver 4.0 software for analyze and normalize the raw data. Raw data was processed following standard procedure to obtain the normalized data. Background correction was performed by subtracting the mean+two standard deviations of negative controls as a cut-off. Next, each value is multiplied by two normalization factor which are obtained from positive controls and spikes. Low expressed miRNAs were excluded from the analysis.

### cDNA Synthesis and RT-qPCR

Of the identified miRNA profile, five miRNAs (miRNA-126-3p, miRNA-191-5p, miRNA-223-3p, miRNA-363-3p and miRNA-495-3p) were selected for validation. The validation of miRNAs expression in serum was performed by reverse transcription and real-time quantitative RT-PCR (Qiagen). Shortly, 1 μl of RNA eluate was reverse transcribed in 10 μl reaction using the miRCURY^®^ LNA^®^ RT Kit (Qiagen) according to the manufacturer’s instructions. The cDNA was diluted 30-fold, 3 μl of this diluted cDNA was used as a template and assayed in 10 μl PCR reactions according to the protocols of the miRCURY^®^ LNA^®^ miRNA PCR Assay (Qiagen). The amplification was performed in a LightCycler^®^ 96 Real-Time PCR System (Roche). qPCR thermocycling conditions were as follows: 95°C for 120 s, followed by 45 cycles of 95°C for 10 s and 60°C for 1 min. Melt curve analysis was performed between 60 and 95°C at a ramp rate of 0.22°C/s. The amplification curves were analyzed using the LightCycler 96 Software 1.1 (Roche). The expression levels of miRNAs were calculated by using 2^–*CT*^ and reciprocal ratios were performed [Ratio miR-A/miR-B = log_2_ (2^–*CTmiR–A*^/2^–*CTmiR–B*^)], as previously described (Pérez-Sánchez et al., 2018).

### Monocytes Subpopulations

Immunolabeling and flow cytometry were performed in whole blood to avoid centrifugation and washing steps which can lead to artifactual platelet activation. To identify CD14+CD16++ monocyte subpopulation, fresh blood (100 μl) were incubated with peridinin chlorophyll protein (PerCP)-conjugated monoclonal anti-CD14 (M5E2), fluorescein isothiocyanate (FITC)-conjugated anti-CD16 (3G8) and isotype matched controls for 20 min at RT in the dark. Both antibodies and the appropriate isotype controls were purchased from BD Biosciences (San Jose, CA, United States). Thereafter, red cells were lysed by the addition of 500 μl of FACS-Lysing solution (Becton Dickinson) for 15 min at RT in the dark. At last, the samples were fixed with 500 μl of CellFix (Becton Dickinson). Promptly, stained cells were acquired on a FACSCalibur flow cytometer (BD Biosciences).

An acquisition threshold was set in forward scatter (FSC) parameter, and unwanted events like platelets, dead cells and debris were not recorded. Monocytes were identified based on their FSC and side scatter (SSC) characteristics. For each sample a minimum of 2500 monocytes were acquired to low flow speed. Post-acquisition analysis was performed using CellQuest (BD Biosciences). The percentage of CD14++CD16-/dim and CD14+CD16++ monocytes was calculated by subtracting non-specific staining, as identified in the isotype control histogram.

### Isolation and Determination of Microvesicles in Plasma

The International Society of Extracellular Vesicles (ISEV) recommends isolating microvesicles after depletion of platelets, cells and large apoptotic bodies by one or more centrifugation steps. Plasma samples were thawed at RT and platelet-free plasma was obtained by centrifugation at 1500 × *g* for 20 min. Then, the supernatant was recovered and centrifuged at 18000 × *g* for 2 min to separate microvesicles (Robert et al., 2009; Alique et al., 2017).

MV were resuspended and incubated with a solution of Annexin V conjugated with phycoerythrin (PE) fluorescein in the presence of CaCl_2_ (5 mM), peridinin chlorophyll protein (PerCP)-conjugated monoclonal anti-CD14 (M5E2) and fluorescein isothiocyanate (FITC)-conjugated anti-CD16 (3G8). As a control for Annexin V labeling, a sample with annexin V conjugated with fluorescein was established using a solution free of CaCl_2_. Labeling was considered optimal if CaCl_2_-labeled sample measurement events were clearly distinguishable from background and CaCl_2_-free staining as well as from isotype controls. Isotype controls were included as negative controls for the CD14 and CD16 labeling. An equal volume of Flow-Count calibration beads (Beckman Coulter, Brea, CA, United States) was added to measure the number of events per microliter. Fluorescence-activated cell sorter analysis was performed on a Cytomics FC 500 flow cytometer (Beckman Coulter) using CXP (Beckman Coulter). MV were defined as particles between 100-1000 nm in size that exhibited significantly more Annexin V fluorescence than their negative controls. Results were expressed as number of Log Annexin V + (total microvesicles), Log CD14 + Annexin V + (monocytes microvesicles) and Log CD14 + Annexin V + CD16 + (proinflammatory monocyte microvesicles) MV/μl plasma.

Before the sample acquisition, the samples were subjected to a separate and combined labeling reaction using all reactive (MAb, Annexin V, and the appropriate negative controls) to compensate for the fluorescence using compensation tools on the flow cytometer. An MV’s gates were established on the Cytomics FC 500 in preliminary standardization experiments using a blend of size-calibrated beads (Beckman Coulter) with diameters of 0.3, 0.5 and 1.0 μm. The upper and outer limits of the MV’s gate were established just above the size distribution of the 1 μm beads in the forward- and side-scatter area (FSC-A and SSC-A, respectively), settings (log scale) to best cover a wide size range. The lower limit was the noise threshold of the instrument (SSC-A), limiting high background noise. Fifty microliters of counting beads with an established concentration close to 1500 beads/μl (Flow Count Fluorospheres; Beckman-Coulter) was added to each sample in order to express microvesicles counts as absolute numbers per microliter of MV. Data collection was not initialized until the count rate of the Flow Count Fluorospheres was stabilized. Samples run on the FC500 instrument were analyzed at the lowest speed with a maximum acquisition rate of 3000 events/seconds. The absolute number of MV was calculated as the following: (MV counted × standard beads/liter)/standard beads counted (Flow-Count; Beckman Coulter). CXP Acquisition and CXP Analysis software packages (Beckman-Coulter) were used for data acquisition and analysis, respectively. The values of total MV (Log Annexin V+), monocytes MV (Log CD14+Annexin V+) and proinflammatory monocytes MV (Log CD14+Annexin V+CD16+) are expressed by the “log”.

### Cell Cultures

#### Human Leukemia Monocytic Cell Line

Human leukemia monocytic cell line (THP-1) was purchased from Sigma-Aldrich (San Luis, Missouri, United States). Cells were cultured in 1640 media containing 15% (v/v) fetal bovine serum and antibiotics (100 U/ml penicillin and 100 μg/ml streptomycin) (Hyclone) at 37°C in an atmosphere of 5% CO_2_. THP-1 cells need cell-to-cell contact to grow. Therefore, THP-1 were seeded at a density around 5 × 10^5^-1 × 10^6^ cells/ml in low attachment 25 cm^2^ rectangular canted cell culture flasks and kept vertically in the incubator.

#### Preparation of Mixed Uremic Toxins

Urea (1200 μg/ml), creatinine (60 μg/ml), oxalic acid (5 μg/ml), indole-3-acetic acid (3.5 μg/ml), indoxyl sulfate (25 μg/ml), and homocysteine (2.7 μg/ml) were dissolved in water. Uric acid (80 μg/ml) being water insoluble was dissolved in 1M NaOH and p-cresol (10 μg/ml) was dissolved in DMSO. These values are the median concentrations observed in uremic patient (Gao et al., 2015).

#### THP-1 and Uremic Medium

To assess the effect of uremic media, THP-1 were incubated with or without mixed uremic toxins for 48 h at 2% FBS to keep the cell in quiescence state.

#### THP-1 Derived Microvesicles Isolation and Quantification

Microvesicles from the culture medium of uremic toxins treated and untreated THP-1 were isolated by ultracentrifugation as previously described by other authors with slight modification (Abbasian et al., 2015). Briefly, the media was centrifuged (Heraeus Labofuge 400R) at 409 × *g* for 5 min at 4°C to remove any intact cells, followed by centrifugation at 789 × *g* for 10 min at 4°C to remove cell debris. The media was then transferred to ultracentrifuge 25 × 89 mm polypropylene tubes (Beckman Coulter, Brea, CA, United States) and centrifuged at 18000 × *g* for 90 min at 10°C in an Optima XPN-100 ultracentrifuge with 70Ti rotor (Beckman Coulter). The MV were sediment owing to relative centrifugal forces. The supernatant containing MV-free media was removed and the pellets containing MV were resuspended in phosphate buffered saline (PBS). Flow cytometry (FC500 Series, Beckman Coulter) was used to determine the quantity of microvesicles secreted by the THP-1 cell in the presence of uremic toxins. Fifty microliters of counting beads with an established concentration close to 1500 beads/μl (Flow Count Fluorospheres; Beckman-Coulter) was added to each sample in order to express microvesicles counts as absolute numbers per microliter of MV. Data collection was not initialized until the count rate of the Flow Count Fluorospheres was stabilized. Samples run on the FC500 instrument were analyzed at the lowest speed with a maximum acquisition rate of 3000 events/seconds. Absolute values of MV were calculated using the following formula: (MV counted × standard beads/liter)/standard beads counted (FlowCount, Beckman Coulter). CXP Acquisition and CXP Analysis software packages (Beckman-Coulter) were used for data acquisition and analysis, respectively. Results were expressed as the number of MV per microliter of culture medium. MV derived from un-treated THP-1 was defined as (cnMV) and uremic toxins-treated THP-1 was defined as uremic toxins MV (txMV).

#### Vascular Smooth Muscle Cells

Human aortic vascular smooth muscle cells (VSMC) were obtained from Clonetics (Lonza Walkersville Inc., Walkersville, MD, United States). Cells were cultured in DMEM supplemented with FBS (20%) (BioWhittaker, Verviers, Belgium), sodium pyruvate (1 mM), glutamine (4.5 g/l), penicillin (100 U/ml), streptomycin (100 mg/ml) and HEPES (20 mM) at 37°C in a humidified atmosphere with 5% CO_2_. Cells were used after the 5^th^ passage.

For studies with VSMC, cells were grown in 6-well plates. When the cells in culture reached 80% confluence, the treatment was started. Three experimental groups were studied: one group of MV-free cells (VSMC) and two groups of cells treated with MV (at a concentration of 10^5^ MV/ml) obtained from the culture medium of mixed uremic toxins-treated (VSMC + txMV) or untreated THP-1 (VSMC + cnMV). The cultures were kept at 37°C in a humid atmosphere and 5% CO_2_ with 2% FBS culture medium for 5 days. The treatment was renewed every 2 days.

#### Cyclin D1, BMP2 and miRNA-223-3p Expression in VSMC Treated With Microvesicles

Total RNA was extracted with Tri-Reagent^TM^ (Sigma, St. Louis, MO, United States) and quantified by spectrophotometry (NanoDrop^®^ ND-1000 UV-Vis Spectrophotometer; NanoDrop Technologies, Wilmington, DE, United States). The mRNA levels of bone morphogenetic protein 2 (BMP2) and cyclin D1 (CycD1) were determined by quantitative real-time RT-PCR (LightCycler^®^ 96, Roche Diagnostics, Basel, Switzerland). A SensiFAST SYBR, NO-ROX ONE-STEP (Bioline) was used to quantify mRNA expression levels. mRNA expression was expressed as a value normalized to levels of GAPDH mRNA (Table 1).

**TABLE 1:** Primer sequences used for RT-PCR.

Gene simbol	Gene name	Forward	Reverse
BMP2	Bone morphogenetic protein 2	*5′-AGG-AGG-CAA-AGA-AAA-GGA-ACG-GAC-3′*	*5′-GGA-AGC-AGC-AAC-GCT-AGA-AGA-CAG-3′*
CycD1	Cyclin D1	*5′-CCG-AGG-AGC-TGC-TGC-AAA-TGG-A-3′*	*5′-ATG-GAG-GGC-GGA-TTG-GAA-ATG-AAC-3′*
GAPDH	Glyceraldehyde-3-Phosphate Dehydrogenase	*5′-TGA-TGA-CAT-CAA-GAA-GGT-GGT-GAA-G-3′*	*5′-TCC-TTG-GAG-GCC-ATG-TGG-GCC-AT-3′*

miRNA-223-3p expression in VSMC was performed by reverse transcription and real-time quantitative RT-PCR (Qiagen). Firstly, 1 μl of RNA eluate was reverse transcribed in 10 μl reaction using the miRCURY LNA RT Kit (Qiagen) according to the manufacturer’s instructions. The cDNA was diluted 30-fold, 3 μl of this diluted cDNA was used as a template and assayed in 10 μl PCR reactions according to the protocols of the miRCURY LNA miRNA PCR Assay (Qiagen). The amplification was performed in a LightCycler 96 Real-Time PCR System (Roche).

#### Analysis of Senescence-Associated β-Galactosidase Activity in VSMC Treated With Microvesicles

Senescence-associated β-galactosidase (SA-β-gal) staining was performed using the Senescence-Galactosidase Staining Kit (MBL International Corporation, Catalog #JM-K320-250) according to the manufacturer’s protocol. In brief, cells were washed with 2 ml PBS and fixed in 2 ml fixing solution for 10 min at RT. Next, the cells were washed once in PBS to remove the fixing solution and incubated in freshly prepared SA-β-gal at 37°C overnight without CO_2_. Afterward, SA-β-gal-positive cells (senescent cells) were identified as blue-stained cells under standard light microscopy (20X) (OPTIKA Microscopes, Ponteranica, BG, Italy) and quantified with the ImageJ analysis software^1^.

### Statistical Analysis

The results are expressed as means ± SEM. Continuous variables were tested for normal distribution with the use of the Kolmogorov–Smirnov test. Chi square test was used for categorical data which were expressed as percentages. Non-parametric comparisons of group differences, such as Mann-Whitney and Kruskal–Wallis tests were used for quantitative variables where appropriate. On the contrary, parametric comparisons were performed using the one-way ANOVA with Bonferroni corrections for multiple comparisons. Correlation analysis was performed between inflammatory markers (CD14+CD16++ and CD14+Annexin V+CD16+MV) and miRNAs ratios. Separately, Pearson or Spearman test where appropriate. SPSS statistics software 15.0 (SPSS Inc., Chicago, IL, United States) and the GraphPad Prism 5.0 (GraphPad Software, La Jolla, CA, United States) were used to perform all the statistical analysis. A *p*-value < 0.05 was considered statistically significant. Agglomerative cluster of genes was performed using nSolver 4.0 analysis software.

## Results

### Characteristics of the Study Population

Demographic characteristics and biochemical parameters of CKD patients included in the present study are shown in Table 2. The mean age of CKD patients (*n* = 45) was 60.9 ± 2.8 years, and the study population comprised 22 men and 23 women, with no significant difference observed among groups with respect to age and gender. Levels of creatinine, BUN, PTH and phosphate were statistically higher in the CKD4-5 and HD groups compared with the KT group. Moreover, serum albumin levels were significantly decreased in HD with respect to CKD4-5 and KT groups. For the rest of the biochemical parameters no significant differences were observed between groups.

**TABLE 2:** Demographic and biochemical characteristics of the 45 patients with chronic kidney disease included in the study.

	Elderly patients	CKD4-5	Hemodialysis	Kidney Transplantation
Number of patients/groups	10	17	11	17
Age, years	80.2 ± 1.34^&,*,#^	65.2 ± 2.7	58.0 ± 3.3	59.7 ± 2.4
Gender (% male)	30.0	47.0	63.6	41.2
eGFR (ml/min/m^2^)	n.a.	14.0 ± 1.7^#^	n.a.	45.0 ± 4.0
Creatinine, mg/dl	0.8 ± 0.01^&,*^	4.2 ± 0.3^*,#^	8.3 ± 0.8	1.5 ± 0.1^*^
BUN, mg/dl	19.1 ± 2.9^&,*^	70.9 ± 5.5^#^	60.8 ± 5.3	33.8 ± 2.8^*^
PTH, pg/ml	n.a.	401.5 ± 63.1^#^	589.1 ± 161.9	148.5 ± 39.9^*^
Phosphate, mg/dl	n.a.	4.6 ± 0.4^#^	4.2 ± 0.4	3.2 ± 0.1
Hemoglobin, gr/dl	n.a.	11.9 ± 0.4	12.4 ± 0.3	12.3 ± 0.3
Albumin, gr/dl	n.a.	4.2 ± 0.1^*^	3.6 ± 0.1	4.2 ± 0.1^*^
Glucose, mg/dl	113.1 ± 12.4	103.0 ± 5.4	111.8 ± 16.9	97.2 ± 6.0
CRP, mg/l	n.a.	6.9 ± 2.2	6.0 ± 2.4	4.8 ± 2.9

The data were analyzed using an ANOVA test and Post hoc Bonferroni to evaluate statistical significance between groups. If the normality or equal variance test was violated, a comparison was made using the non-parametric Mann–Whitney U-test. Chi square test was used for categorical data. The results are represented as the means ± SEM. ^&^p < 0.05 vs. CKD4-5; *p < 0.05 vs. hemodialysis; #p < 0.05 vs. kidney transplantation; not applicable (n.a.).

### miRNAs Profile in CKD Patients

**FIGURE 1:**
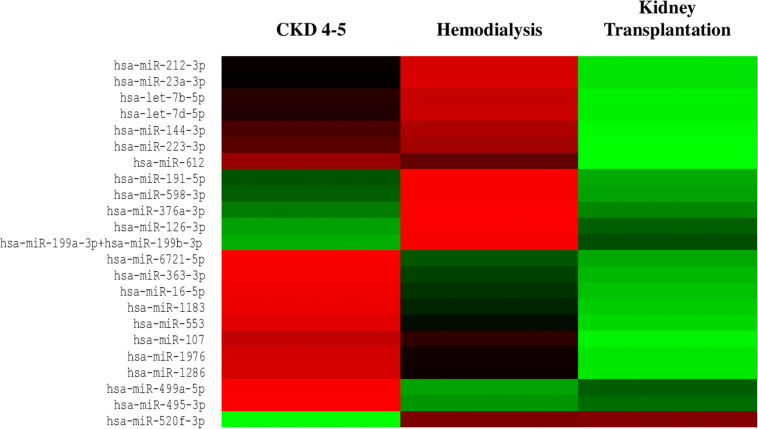
Heat map of differentially expressed miRNAs from serum samples of CKD patients. Circulating miRNAs expression was downregulated in CKD4-5 and HD patients whereas was upregulated in KT patients. Red indicates downregulated miRNAs expression and green indicates upregulated miRNAs expression. *n* = 4 each group.

**FIGURE 2:**
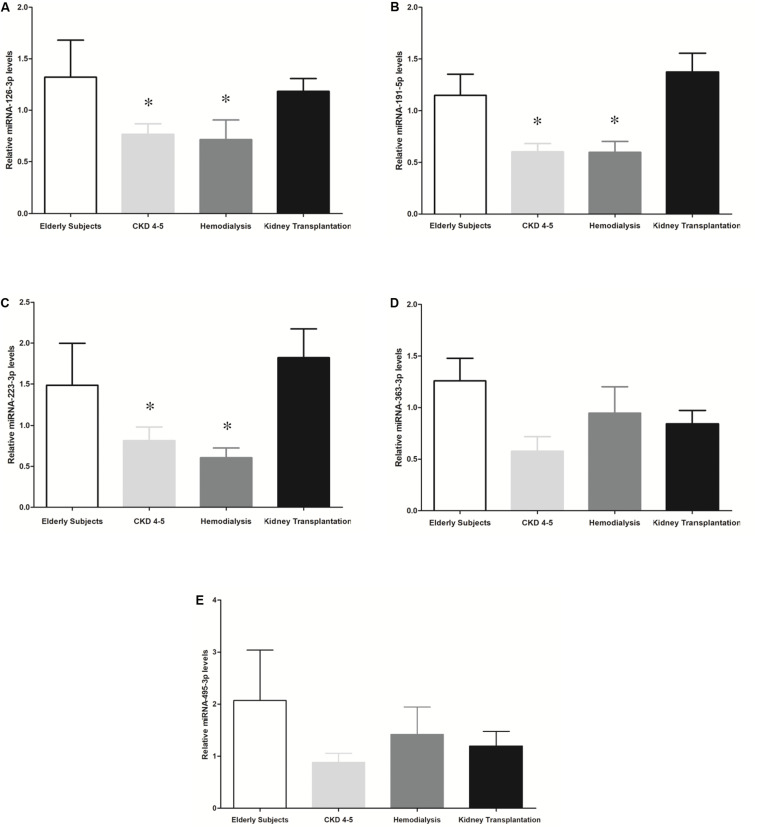
Expression relative miRNAs levels in CKD patients and elderly subjects. The results are represented as the means ± SEM. Relative expression levels of circulating: **(A)** miRNA-126-3p; **(B)** miRNA-191-3p; **(C)** miRNA-223-3p; **(D)** miRNA-363-3p and **(E)** miRNA-495-3p are shown. The data were analyzed using an ANOVA test and *Post hoc* Bonferroni to evaluate statistical significance between groups. If the normality or equal variance test was violated, a comparison was made using the non-parametric Mann–Whitney U-test. *p*-value < 0.05 was considered on the borderline of statistical significance. *p < 0.03 vs. kidney transplantation.

We exhaustively screened the expression of circulating miRNAs in a small group of random samples selected from all available groups. Those miRNAs with a low copy number were removed from the analysis. Accordingly, 25 out of 827 miRNAs were differentially expressed in the different CKD stages (Figure 1). To validate the results obtained in the identification phase, we analyzed the expression levels of 5 miRNAs (miRNA-126-3p, miRNA-191-5p, miRNA-223-3p, miRNA-363-3p and miRNA-495-3p) in all samples by RT-PCR analysis. We observed a reduced expression levels of miRNAs-126-3p, -191-5p and -223-3p in CKD4-5 (1. 54-, 2.29- and 2.24-fold, respectively) and HD (1. 65-, 2.30- and 3.0-fold, respectively) patients as compared to KT patients (Figures 2A–C). No significant differences in miRNA-363-3p and miRNA-495-3p expression levels were observed between groups (Figures 2D,E).

**FIGURE 3:**
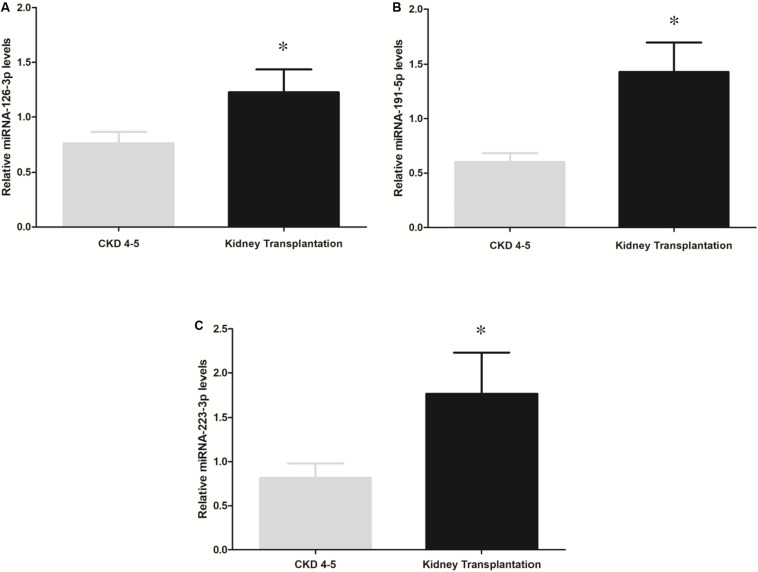
Comparison of miRNAs expression between the CKD4-5 and KT patients with a similar eGFR [“KT (CKD4-5 equivalent)]”, eGFR between 6 and 29 [ml/min/1.73m^2^]). **(A)** miRNA-126-3p, **(B)** miRNA-191-5p and **(C)** miRNA-223-3p. The data were analyzed using student’s *t*-test. Data are depicted as means ± SEM. *p*-value < 0.05 was considered on the borderline of statistical significance. *p ≤ 0.02.

To determine whether disease status may determine miRNAs expression profile, we analyzed differences in miRNAs expression levels between CKD4-5 and KT patients with similar eGFR (6-29 ml/min). However, miRNA-126-3p (1.6-fold, *p* = 0.03), miRNA-191-5p (2.17-fold, *p* < 0.001) and miR-223-3p (2.37-fold, *p* = 0.02) remained significatively reduced in CKD4-5 patients as compared to KT patients matched by eGFR (Figure 3).

**FIGURE 4:**
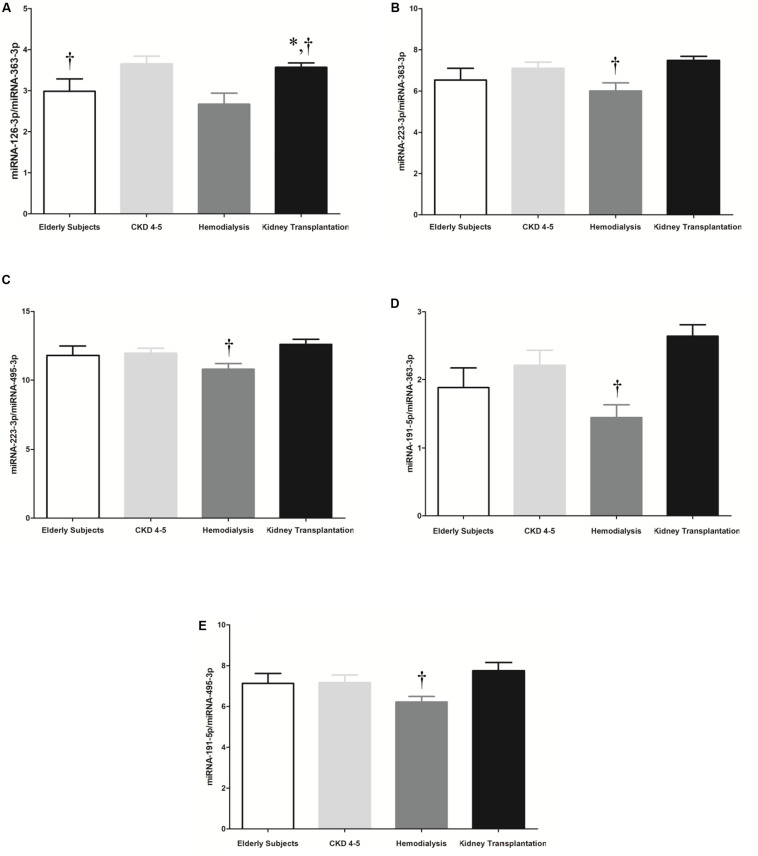
Selected 5 microRNA ratios were analyzed in the whole cohort, including 45 CKD patients and 10 elderly subjects. **(A)** miRNA-126-3p/miRNA-363-3p, **(B)** miRNA-223-3p/miRNA-363-3p, **(C)** miRNA-223-3p/miRNA-495-5p, **(D)** miRNA-191-5p/miRNA-363-3p and **(E)** miRNA-191-5p/miRNA-495-5p. The data were analyzed using an ANOVA test and *Post hoc* Bonferroni to evaluate statistical significance between groups. *p*-value < 0.05 was considered on the borderline of statistical significance. *p < 0.05 vs. CKD4-5; ^†^*p* < 0.05 vs. kidney transplantation.

It has been shown that the combination of miRNAs improves their predictive potential as biomarkers of disease progression (Sharova et al., 2016). Thus, reciprocal ratios of the miRNAs analyzed were performed. Five miRNA ratios were found to be significantly different between groups (*p* < 0.05), including miRNA-126-3p/miRNA-363-3p, miRNA-223-3p/miRNA-363-3p, miRNA-223-3p/miRNA-495-5p, miRNA- 195-5p/miRNA-363-3p and miRNA-191-5p/miRNA-495-5p (Figure 4). We focused on the miRNA-126-3p/miRNA-363-3p, miRNA-223-3p/miRNA-363-3p, miRNA-191-5p/miRNA-363-3p, miRNA-495-5p/miRNA-363-3p which showed a strong association with proinflammatory monocytes and monocyte-derived MV (CD14+Annexin V+CD16+MV) (Table 3).

**TABLE 3:** CD14+CD16++ and CD14+Annexin V+CD16+MV correlation analysis.

	Correlation coefficient	*p*-value
CD14+CD16++ vs. miRNA-126-3p/miRNA-363-3p	–0.63	< 0.0001
CD14+CD16++ vs. miRNA-223-3p/miRNA-363-3p	–0.53	0.0003
CD14+CD16++ vs. miRNA-495-5p/miRNA-363-3p	–0.57	< 0.0001
CD14+CD16++ vs. miRNA-191-5p/miRNA-363-3p	–0.57	< 0.0001
CD14+Annexin V+CD16+MV vs. miRNA-126-3p/miRNA-495-5p	0.48	0.005
CD14+Annexin V+CD16+MV vs. miRNA-126-3p/miRNA-191-5p	–0.47	0.005
CD14+Annexin V+CD16+MV vs. miRNA-223-3p/miRNA-495-5p	0.45	0.01
CD14+Annexin V+CD16+MV vs. miRNA-495-5p miRNA-363-3p	–0.42	0.01
CD14+Annexin V+CD16+MV vs. miRNA-191-5p/miRNA-363-3p	–0.35	0.04
CD14+Annexin V+CD16+MV vs. miRNA-495-5p/miRNA-191-5p	–0.60	0.0003

### Monocyte Subpopulations in CKD Patients

Several studies show that mature and/or activated CD14+CD16++ blood monocytes are associated to progression of renal disease. To identify whether this monocytes subset is differentially represented in CKD patients, we applied two-color flow cytometry. Monocyte subsets were defined as CD14++ CD16-/dim (classical monocytes), and CD14+CD16++ (proinflammatory monocytes).

**FIGURE 5:**
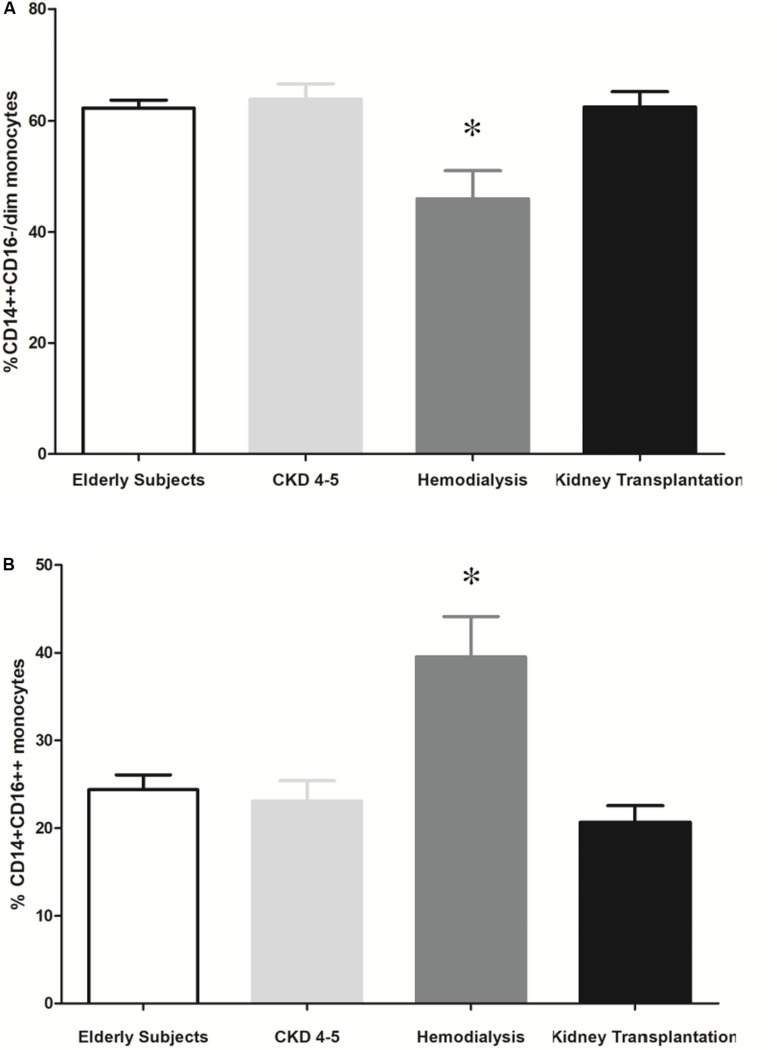
Representative flow cytometry monocytes subsets. The results are represented as the means ± SEM. Percentage of **(A)** CD14++ CD16-/dim and **(B)** CD14+CD16++ monocytes in elderly patients, CKD4-5, hemodialysis and kidney transplantation patients. The data were analyzed using an ANOVA test and *Post hoc* Bonferroni to evaluate statistical significance between groups. *p*-value < 0.05 was considered on the borderline of statistical significance. *p < 0.05 vs. all the groups.

Blood leukocyte subpopulation of CKD patients are shown in Table 4. CKD4-5 patients showed increased total blood leukocytes number respect to HD and KT patients. Specifically, eosinophils number was higher in CKD4-5 patients as compared with HD and KT patients and eosinophils number was lower in KT patients than in HD patients. In relation to the distribution of monocyte subsets, our results show that the percentage of circulating CD14++ CD16-/dim was lower in HD patients (45.9 ± 5.1%) compared with elderly (62.3 ± 1.4%, *p* = 0.007), CKD4-5 (63.9 ± 2.8%, *p* = 0.001) and KT patients (62.5 ± 2.8%, *p* = 0.004) (Figure 5A). Conversely, the percentage of CD14+CD16++ monocytes was significantly increased in HD patients (39.5 ± 4.6%) compared with elderly (24.4 ± 1.7%, *p* < 0.05), CKD4-5 (23.1 ± 2.3%, *p* < 0.05) and KT patients (20.7 ± 1.9%, *p* < 0.001). No significant differences in monocytes subpopulation were observed between CKD4-5 and KT groups or with elderly subjects in monocytes subpopulation (Figure 5B).

**TABLE 4:** Blood leukocyte subpopulations of the 45 patients with chronic kidney disease.

	Elderly patients	CKD4-5	Hemodialysis	Kidney Transplantation
Leukocytes (10^3^ per μ l)	8.92 ± 0.97^*,#^	8.15 ± 0.63^*,#^	6.30 ± 0.93	5.83 ± 0.47
Neutrophils (10^3^ per μ l,%)	6.62 ± 0.82 (74.22)^*,#^	5.12 ± 0.53 (62.82)	4.20 ± 0.88 (66.67)	3.64 ± 0.35 (62.43)
Lymphocytes (10^3^ per μ l,%)	1.59 ± 0.26 (17.82)	1.94 ± 0.24 (23.80)	1.38 ± 0.10 (21.90)	1.55 ± 0.16(26.59)
Eosinophils (10^3^ per μ l,%)	0.12 ± 0.03 (1.34)^&^	0.30 ± 0.06 (3.68)^*,#^	0.14 ± 0,03 (2.22)	0.05 ± 0.02 (0.86)^*^
Monocytes (10^3^ per μ l,%)	0.46 ± 0.05 (5.15)	0.54 ± 0.05 (6.62)	0.38 ± 0.04 (6.03)	0.40 ± 0.06 (6.86)
Basophils (10^3^ per μl,%)	0.03 ± 0.005 (0.33)	0.04 ± 0.01 (0.49)	0.03 ± 0.01 (0.48)	0.05 ± 0.01 (0.86)

The data were analyzed using an ANOVA test and Post hoc Bonferroni to evaluate statistical significance between groups. If the normality or equal variance test was violated, a comparison was made using the non-parametric Mann–Whitney U-test. The results are represented as the means ± SEM. ^&^p < 0.05 vs. CKD4-5; *p < 0.05 vs. hemodialysis; ^#^p < 0.05 vs. kidney transplantation.

### Microvesicles Levels in CKD Patients

**FIGURE 6:**
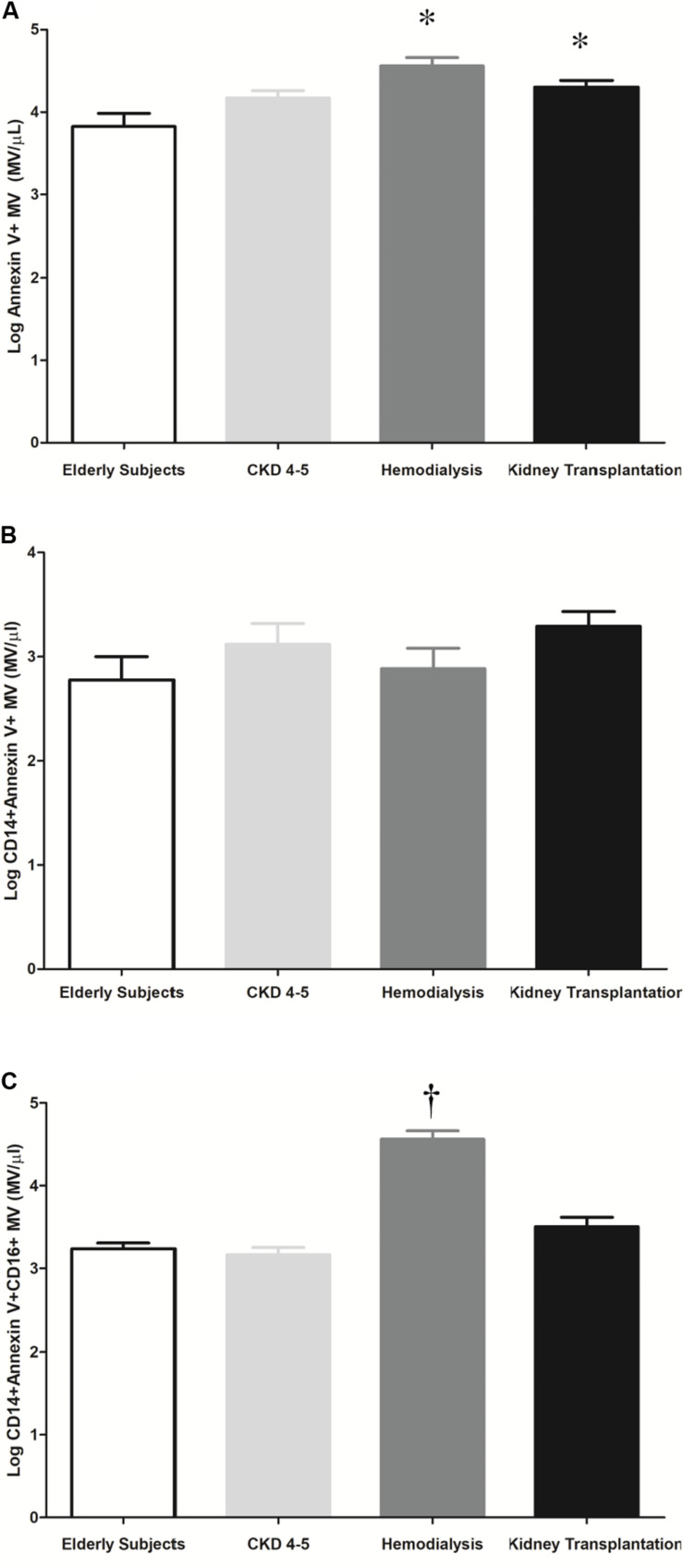
Representative histogram and quantification of microvesicles (MV/μl) in plasma by flow cytometry. The results are represented as the means ± SEM. **(A)** Number of microvesicles per microliter (Log Annexin V + MV) in elderly subjects, CKD4-5, hemodialysis and kidney transplantation patients. **(B)** Number of microvesicles per microliter (Log CD14 + Annexin V + MV) in elderly subjects, CKD4-5, hemodialysis and kidney transplantation patients. **(C)** Number of microvesicles per microliter (Log CD14 + Annexin V + CD16 + MV) in elderly patients, CKD4-5, hemodialysis and kidney transplantation patients. The data were analyzed using an ANOVA test and *Post hoc* Bonferroni to evaluate statistical significance between groups. *p*-value < 0.05 was considered on the borderline of statistical significance. *p ≤ 0.03 vs. elderly subjects; ^†^*p* < 0.001vs. all groups.

MV are produced and released into the circulation as a direct response to cell stimulation. We observed higher total microvesicles number in HD and KT patients as compared to elderly subjects [4.6 ± 0.1 vs. 3.8 ± 0.2 (MV/μl), *p* < 0.001, (HD vs. elderly); and 4.3 ± 0.1 vs. 3.8 ± 0.2 MV/μl, *p* ≤ 0.03, (KT vs. elderly)] (Figure 6A). No differences we observed in monocytes microvesicles (CD14+ Annexin V+MV) between the study groups (Figure 6B). However, in HD patients, we observed a significant increase in the plasma concentration of microvesicles derived from proinflammatory monocytes (CD14+ Annexin V+CD16+MV) as compared with elderly, CKD4-5 and KT patients (4.6 ± 0.1 vs. 3.2 ± 0.1, 3.2 ± 0.1, and 3.5 ± 0.1 MV/μl, *p* < 0.001, respectively), (Figure 6C).

### Correlation Between Proinflammatory Monocytes and Microvesicles Derived From Proinflammatory Monocytes

**FIGURE 7:**
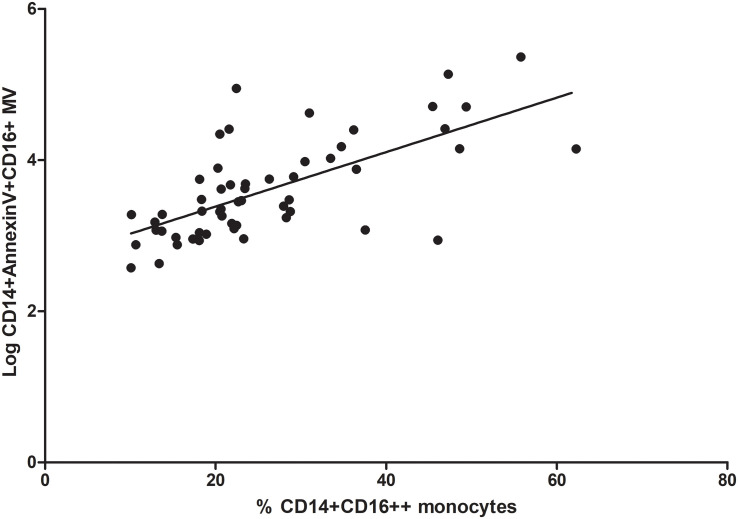
Correlation between proinflammatory monocytes (% CD14+CD16++) and microvesicles (Log CD14 + Annexin V + CD16 + MV).

Interesting, we observed a positive correlation between the percentage of CD14+CD16++ monocytes (proinflammatory monocytes) and microvesicles derived from proinflammatory monocytes (CD14 + Annexin V + CD16 + MV) (rho correlation Spearman = 0.66; *p* < 0.0001 Figure 7).

### Microvesicles Derived From Uremic Toxins-Treated THP-1 Induces Senescence and Osteogenic Markers Expression in VSMC

**FIGURE 8:**
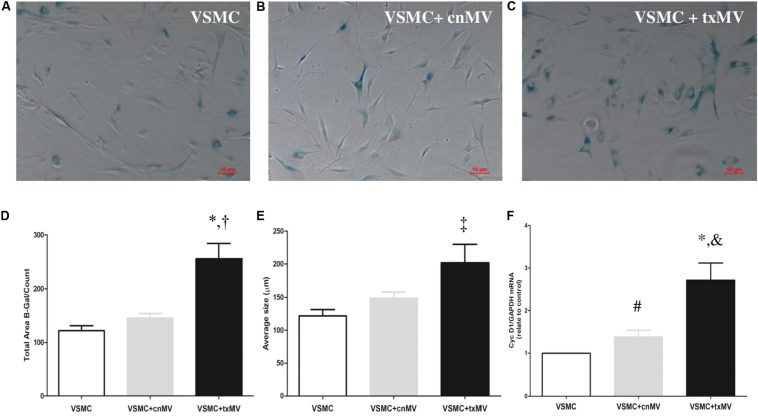
Microvesicles derived from uremic toxins-treated THP-1 induces senescence of vascular smooth muscle cells. Senescent-associated β-Gal activity staining of different of MV. **(A–C)** Representative images of inverted optical microscopy of the senescence studies of **(A)** control VSMC, **(B)** VSMC treated with cnMV and **(C)** txMV. **(D)** Quantification Total Area β-Gal/count and **(E)** Average size (μm) of β-Gal positive cells. **(F)** Expression level of cyclin D1was measured in VSMC. The data were analyzed using an ANOVA test and *Post hoc* Bonferroni to evaluate statistical significance between groups. If the normality or equal variance test was violated, a comparison was made using the non-parametric Mann–Whitney U-test. Data are the means ± SEM of six independent experiments. *p < 0.001 vs. VSMC; ^†^*p* < 0.001 vs. VSMC + cnMV; ^‡^*p* = 0.009 vs. VSMC; ^ #^*p* = 0.015; ^&^*p* = 0.01 vs. VSMC + cnMV.

To understand the molecular mechanism involved in senescence and osteogenic differentiation of VSMC, these cells were stimulated with MV isolated from uremic toxins-treated THP-1 monocytes (txMV). As shown in Figures 8A–C, increased senescence was observed in VSMC treated with txMV. In this sense, there was an increase in the total β-galactosidase labeled area in VSMC treated with txMV with respect to VSMC (2.11-fold) and VSMC + cnMV (1.75-fold) (*p* < 0.001) (Figure 8D). In addition, we observed an increase in average size in txMV-treated VSMC (1.66-fold) with respect to control VSMC (*p* = 0.009) (Figure 8E). No differences were observed between cnMV-treated VSMC and control ones. In parallel, we measured expression level of CycD1, a marker of replicative senescence, in VSMC. The miRNA expression of CycD1 was elevated in cells treated with cnMV (1.38-fold, *p* = 0.015) and txMV (2.71-fold, *p* = 0.001) compared with control cells. In cells treated with txMV, the CycD1 mRNA level was 1.96-fold greater than in cells treated with cnMV (*p* = 0.01) (Figure 8F).

**FIGURE 9:**
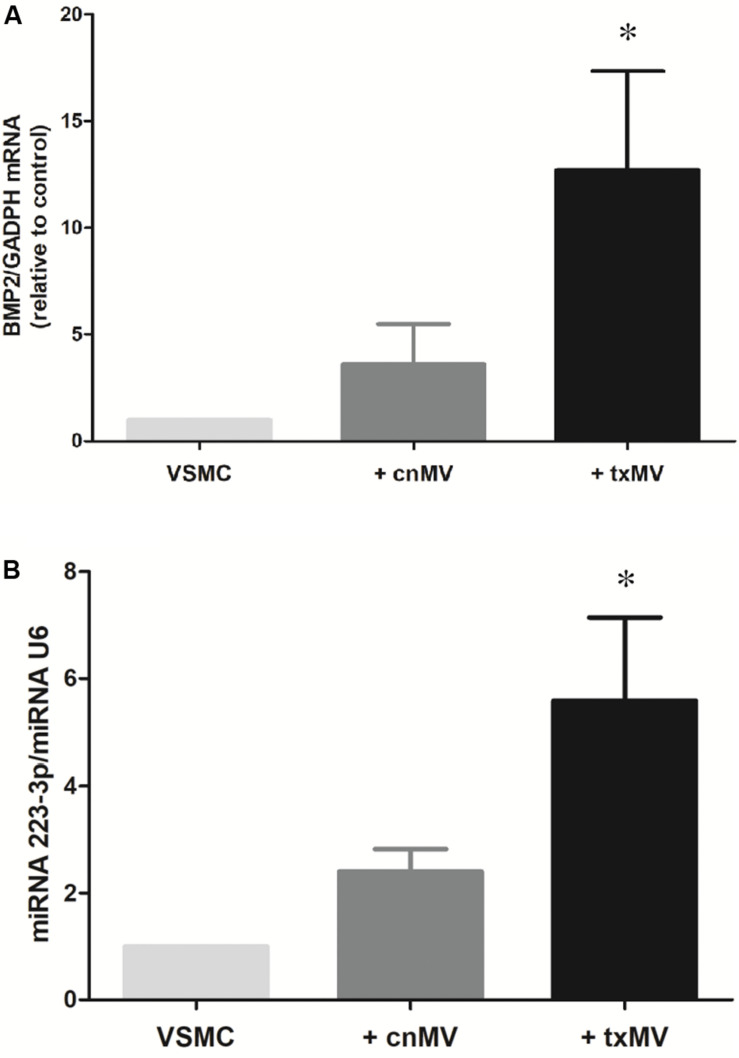
Expression levels of BMP2 and miRNA-223-3p in vascular smooth muscle cells (VSMC). Histogram of **(A)** BMP2 mRNA and **(B)** miRNA-223-3p levels were measured in VSMC. txMV induces differential expression of BMP2 and miRNA-223-3p in VSMC. The data were analyzed using an ANOVA test and *Post hoc* Bonferroni to evaluate statistical significance between groups. Data are the means ± SEM of six independent experiments. *p < 0.02 vs. VSMC.

It is known that bone morphogenetic protein 2 (BMP2) and miR-223-3p regulate important aspects of inflammation and vascular calcification. Thus, to evaluate the role of txMV in the differentiation osteogenic process were measured expression levels of BMP2 and miRNA-223-3p in VSMC. As shown in Figure 9, BMP2 mRNA (10.8-fold) and miRNA-223-3p (5.58-fold) expression were augmented in txMV treated VSMC as compared to control (*p* = 0.02). However, no differences were reported when we analyzed the expression of other miRNAs related to inflammation, (miRNA-126-3p, miRNA-191-5p) (data no shown).

## Discussion

Chronic inflammation is a common feature and a major cause of cardiovascular and other complications of CKD (Yeun et al., 2000; Yamada et al., 2014). In our study, we evaluated the quantitative changes of miRNA-126-3p, miRNA-191-5p and miRNA-223-3p, CD14+C16++ monocytes and microvesicles (total MV and monocytes-derived MV) in patients at various CKD stages. In addition, the effects of microvesicles derived from monocytes on VSMC were studied. Our *in vitro* data show that microvesicles derived from uremic toxins-exposed monocytes (txMV) induce the expression of osteogenic markers and early senescence in VSMC.

miRNAs are differently expressed in CKD patients, playing a key role in pathogenesis and progression of renal disease (Trionfini et al., 2015). In the present study, miRNAs expression profiling analysis identified a set of miRNAs differentially expressed at different stages of CKD. We observed lower expression of miRNA-126-3p, miRNA-191-5p and miRNA-223-3p in CKD4-5 and HD as compared with KT patients. These differences remained significantly different after adjustment by eGFR, indicating that regulation of these miRNAs is independent of kidney function. Our results are in accordance with other authors which have reported that circulating miRNA-126-3p is decreased in ESRD (Fourdinier et al., 2019) and HD patients (Brigant et al., 2017). miRNA-126-3p has an important role in vascular dysfunction, since it enhances endothelial proliferation and endothelization of large vessels, which in turn attenuates atherosclerosis (Davignon and Ganz, 2004; Zernecke et al., 2009; Economou et al., 2015).

Likewise, miRNA-223-3p is implicated in vascular complications that occur during the later stages of CKD (Brigant et al., 2017). Various studies have reported a significantly lower systemic expression of miR-223-3p at later stages of CKD, as well as up-regulation of this miRNA after kidney transplantation (Ulbing et al., 2017; Fourdinier et al., 2019). miR-223 deregulation was recently associated with vascular calcification, osteoclast differentiation, VSMC synthetic phenotype, and inflammation throughout different mechanisms (Taïbi et al., 2014). miRNA-191-5p is known to be dysregulated in several tumors and it has been proposed as a novel diagnostic and therapeutic biomarker (Chen P. et al., 2018). In the same way, other authors found that miRNA-191-5p was significantly downregulated in Alzheimer’s disease (Chen J. et al., 2018) and venous thromboembolism (Starikova et al., 2015). Recently, miRNA-191-5p have been identified as independent biomarkers of CKD in hypertensive patients (Berillo et al., 2020). However, to our knowledge, no previous study has analyzed the role of this miRNA at different stages of CKD. The combination of miRNAs may increase their predictive potential as biomarkers of disease (Boeri et al., 2011; Sheinerman et al., 2013; Sharova et al., 2016; Pérez-Sánchez et al., 2018). In our study, we identified 4 miRNA ratios, differentially expressed, that exhibited significant and positive correlations with clinical features of the disease, particularly the inflammatory status.

Systemic inflammation is a hallmark of CKD and proinflammatory monocyte subset (CD14+CD16++) plays a key role in the development and progression of CVD (Lee et al., 2013). Chronic inflammation state has been related to activation of mononuclear cells in CKD patients (Cohen et al., 1997). CD14+CD16++ monocytes have proinflammatory activity that is associated with an increased risk of atherosclerosis and CVD (Merino et al., 2008). It is known that the percentage of CD14+CD16++ monocytes is increased in CKD patients (Ramírez et al., 2005; Ziegler-Heitbrock, 2007). In earlier studies, it was reported that patients undergoing hemodialysis have substantially higher CD14+CD16++ monocyte number than patients with advanced CKD before initiation of dialysis (Amabile et al., 2005; Ramírez et al., 2005; Ramirez et al., 2007). Consistent with previous results, we have observed a higher percentage of proinflammatory monocytes (CD14+CD16++) and a lower percentage of classical monocytes (CD14++CD16-/dim) in HD patients compared to the other study groups. In our study, elderly patients with normal renal function were included to evaluate the inherent early senescence process that occurs in patients with CKD regardless of age. Probably due to this, no significant differences were found in the percentage of proinflammatory monocytes in patients with CKD4-5 and KT patients compared to elderly subjects. In this line, Merino A et al. noted that the percentage of CD14+CD16++ was similar in healthy elderly subjects compared to patients with CKD (Merino et al., 2011).

MV, a plasma membrane-derived subclass of extracellular vesicles, are produced and released into the circulation as a direct response to cell stimulation. Circulating MV are augmented in CKD, playing a pathological effect on endothelial dysfunction (Amabile et al., 2005), vascular calcification (Faure et al., 2006; Chen et al., 2015; Dickhout and Koenen, 2018; Feng et al., 2020) and cardiovascular mortality (Carmona et al., 2017a). MV may have phosphatidylserine on their outer membrane enabling the use of conjugated annexin V antibodies for their detection (Erdbrügger and Lannigan, 2016), because of this, we have defined circulating microvesicles, whatever their cellular origin, as Annexin V + MV. Our result shown that plasma of CKD patients, mainly HD patients, contained more circulating MV than elderly subject, in agreement with previous studies (Mohandas and Segal, 2010; Carmona et al., 2017a). We expected to find a relevant decrease in circulating MV levels in KT patients, but no differences after kidney graft were observed. This fact may be due to the time elapsed since transplantation was not enough to find significant differences in the total microvesicles levels. Some authors have reported that monocytes microvesicles levels are strongly correlated to the extent of vascular disease and have been related to increased risk for CV morbidity and mortality (Kanhai et al., 2013; Eikendal et al., 2014; Vrijenhoek et al., 2015). In our study, we did not observe statistically significant differences in monocytes microvesicles levels (CD14 + Annexin V + MV) between groups. However, we next analyzed the number of MV exclusively released by proinflammatory monocytes. CD14 + Annexin V + CD16 + MV were increased in CKD patients undergoing HD treatment. Interestingly, our results showed that the levels of circulating CD14 + Annexin V + CD16 + MV correlated positively with the subpopulation of proinflammatory monocytes CD14+CD16 ++. This could partly explain the chronic inflammatory status of our patients, resulting from the uremic environment associated to CKD. We propose that, CD14 + Annexin V + CD16 + microvesicles could be considered as proinflammatory molecules that enhance vascular inflammation. Interestingly, this is the first report that evaluate proinflammatory microvesicles (CD14 + Annexin V + CD16 + MV) in CKD patients, thus, future studies are needed to validate this hypothesis and to identify the specific role of these MV on renal pathophysiology.

In presence of certain stimuli, monocytes secrete extracellular vesicles that promote endothelial inflammation and atherosclerosis progression in both *in vivo* and *in vitro* assays (Aronow, 2017). We generated MV from THP-1 under uremic toxins stimulation to mimic inflammation in CKD and to evaluate its effect on VSMC. Increased expression of the senescent marker β-galactosidase and CycD1, as well as changes in VSMC size were observed in those cells treated with MV derived from uremic toxins-treated THP-1. The increased in VSMC size was in accordance with previous data reporting that senescent human cells showed higher cellular size (Biran et al., 2017; Veitia, 2019). In parallel, we also demonstrated that the senescence observed in the txMV-treated cells was accompanied by an upregulation in the expression of CycD1, a marker of senescence in VSMC (Burton et al., 2007). Of note, it has been suggested that senescent VSMC contribute to cardiovascular dysfunction through induction of vascular calcification (Burton et al., 2010; Greco et al., 2019; Herrmann et al., 2020; Song et al., 2020). In this sense, we observed an increased expression of BMP2 and miR-223-3p in cells in the txMV-treated cells. These results are in line with previous studies, where miRNA-223 was upregulated in VSMC in the presence of inorganic phosphate, a known calcifying uremic toxin (Rangrez et al., 2012; Shan et al., 2015). Another marker directly related to the CVD associated with uremia is BMP2 (Canalis et al., 2003). A previous study by our group showed that microparticles produced by endothelial cells in response to inflammatory stimuli promote a calcifying response, overexpression BMP2, in VSMC (Buendía et al., 2015). Therefore, our combined results suggest that these MV could be involved in the early cellular senescence observed in CKD patients as well as the osteogenic transdifferentation of VSMC.

Our study has several limitations. First, the study was conducted in a single center with a small cohort patient. Therefore, our results should be confirmed in a large sample- and multicenter study. An additional limitation is related to the fact that microvesicles count may be disturbed by possible variations in the cytometer aspiration flow rate during analysis. However, the use of an internal standard such as beads of a known concentration could rule out this possibility.

In conclusion, our data suggest that CKD patients present a specific circulating miRNAs expression profile, which is dependent, in part, on the modality of therapy. In this line, serum levels of circulating miRNAs were associated with the inflammatory state in uremia. Furthermore, microvesicles generated by monocytes treated with uremic toxins induce early senescence and an increase in osteogenic markers (BMP2 and miRNA-223-3p) in VSMC, which could promote vascular damage in CKD. Therefore, microvesicles are much more than a biomarker of endothelial damage and should probably be considered as a therapeutic target to prevent endothelial damage and vascular disease in CKD.

## Data Availability Statement

The authors acknowledge that the data presented in this study must be deposited and made publicly available in an acceptable repository, prior to publication. Frontiers cannot accept a manuscript that does not adhere to our open data policies.

## Ethics Statement

The studies involving human participants were reviewed and approved by the Reina Sofia Hospital Ethics Committee. The patients/participants provided their written informed consent to participate in this study.

## Author Contributions

AC, FG, and PA conceived and designed the research. AC, FG, MJ, and FA performed the experiments. AC, FG, JM, AM-M, and PA analyzed the data and drafted the manuscript. AC, FG, MJ, FA, MA, TO, VN, JM-C, MR, SS, JM, AM-M, and PA interpreted results of the experiments, edited and revised the manuscript, and approved final version of the manuscript. AC and FG prepared the figures. All authors contributed to the article and approved the submitted version.

## Conflict of Interest

The authors declare that the research was conducted in the absence of any commercial or financial relationships that could be construed as a potential conflict of interest.

## Footnotes

1 http://rsb.info.nih.gov/ij/
